# Dosimetric evaluation of bone marrow sparing in proton radiotherapy for cervical cancer guided by MR functional imaging

**DOI:** 10.1186/s13014-022-02175-3

**Published:** 2022-12-14

**Authors:** Xiaohang Qin, Guanzhong Gong, Lizhen Wang, Ya Su, Yong Yin

**Affiliations:** 1 Department of Graduate, Shandong First Medical University, Shandong Academy of Medical Sciences, Jinan, China; 2 Department of Radiation Physics, Shandong Cancer Hospital and Institute, Shandong First Medical University and Shandong Academy of Medical Sciences, Jinan, China

**Keywords:** Pelvic active bone marrow, Bone marrow sparing, Proton therapy, MR functional imaging, Cervical cancer

## Abstract

**Background:**

To segment the pelvic active bone marrow (PABM) using magnetic resonance (MR) functional imaging and investigate the feasibility and dosimetric characteristics of cervical cancer proton radiotherapy for active bone marrow (ABM) sparing.

**Methods:**

We collected CT and MR simulation images of 33 patients with cervical cancer retrospectively. The PBM was contoured on the MRI FatFrac images; the PBM was divided into high-active bone marrow (ABM-_high_) and low-active bone marrow based on the fat content of the PBM. Four radiotherapy plans were created for each patient, which included intensity-modulated photon therapy (IMRT), bone marrow sparing IMRT (IMRT-BMS), intensity-modulated proton therapy (IMPT), and bone marrow sparing IMPT (IMPT-BMS). The dosimetric differences among the four plans were compared.

**Results:**

The ABM-_high_ volume in the enrolled patients accounted for 45.2% of the total ABM volume. The target coverage was similar among the four radiotherapy plans. IMRT-BMS, IMPT, and IMPT-BMS reduced the D_mean_ of ABM-_high_ by 16.6%, 14.2%, and 44.5%, respectively, compared to the D_mean_ of IMRT (*p* < 0.05). IMPT-BMS had the best protective effect on the bone marrow. Compared to IMRT, the volume of ABM-_high_ receiving an irradiation dose of 5–40 Gy decreased by 10.2%, 36.8%, 58.8%, 67.4%, 64.9%, and 44.5%, respectively (*p* < 0.001).

**Conclusions:**

The MR functional imaging technique helped in the grading and segmentation of PABM. MR functional image-guided proton radiotherapy for cervical cancer can achieve optimal BMS.

**Supplementary Information:**

The online version contains supplementary material available at 10.1186/s13014-022-02175-3.

## Background

Concurrent chemoradiotherapy can improve the clinical efficacy of cervical cancer treatment, but hematological toxicity (HT) can considerably decrease the survival time and quality of life of patients [1, 2]. Compared to radiotherapy alone, concurrent chemoradiotherapy affects the systemic immune response, resulting in a decrease in compensatory responses in the bone marrow (BM) outside the pelvis and an increase in the incidence of HT [3–7]. The unavoidable dose of radiation to the pelvic active bone marrow (PABM) in cervical cancer radiotherapy is an important cause of HT. Low-dose irradiation of 10–20 Gy to PBM is the primary cause of HT [8–10]. In our previous study, we found a significant positive correlation between fat content changes in the 5–10 Gy irradiated PBM region and the nadir of lymphocytes and neutrophils during chemoradiotherapy [11]. A meta-analysis showed that bone marrow sparing (BMS) techniques could effectively reduce the bone marrow dose and the incidence of grade 2 or 3 HT by approximately 70% [12].

BM is divided into hematopoietic active bone marrow (ABM) and inactive BM, each accounting for approximately 50%. Approximately half of the ABM is distributed in the pelvic region. A dose of radiation to the ABM during radiotherapy is a major factor in the occurrence of acute HT. Various quantitative and qualitative imaging techniques help to accurately identify active fractions in the BM [13]. The accurate localization and sparing of ABM can reduce HT and improve tolerance to chemotherapy and survival rates [14, 15]. Magnetic resonance (MR) FatFrac imaging can be used to quantitatively analyze the fat content of the BM and can reflect the fat content changes in PBM during cervical cancer radiotherapy [11]. MR functional imaging provides a feasible method for ABM segmentation.

A photon-based BMS plan can effectively reduce the exposed volume of BM. The proton Bragg peak can further concentrate the dose to the target relative to the photon plan. Only a few fields are needed to meet the dose coverage of the target. Proton therapy has great potential for BMS due to its physical dose deposition properties that can effectively protect normal tissues adjacent to the target [16, 17]. Therefore, we used MR functional imaging to segment the PABM. We also analyzed the dosimetric differences between photon and proton BMS radiotherapy plans for cervical cancer. In this study, we determined the feasibility and dosimetric characteristics of MR functional imaging-guided BMS proton radiotherapy for cervical cancer.

## Materials and methods

In total, 33 patients with cervical cancer who received concurrent chemoradiotherapy in Shandong Cancer Hospital from 2019 to 2020 were recruited. All patients received 45–50 Gy/25 fractions of pelvic intensity-modulated photon therapy (IMRT) combined with concurrent cisplatin chemotherapy. Each patient underwent CT and multi-sequence MR simulation scans. CT scans were performed using a Philips 16-slice Brilliance big-bore computed tomography scanner (Philips Medical Systems, Amsterdam, Netherlands) with a 3-mm slice gap thickness and 3-mm slice. The patients were immobilized in the supine position with thermoplastic molds or in the prone position with an abdominal pelvic fixator. Magnetic resonance imaging (MRI) was performed using a 3.0T superconducting MR scanner (Discovery 750w, GE Healthcare, USA) with the same position and fixed device as those used for the CT scans. All patients underwent T1WI, T2fs, and IDEAL IQ sequence scans with 3 mm slice thickness. The IDEAL IQ sequence scans were reconstructed to obtain the fat fraction (FatFrac IDEAL IQ) images.

### Target and BM contouring

The targets and organ at risk (OAR) were contoured on CT and MR fusion registration images. The gross tumor volume (GTV) of the uterus, cervix, parametrium, upper third of the vagina, and locoregional lymph nodes, which included common, internal and external iliac, obturator, and presacral lymph nodes, were all included in the clinical target volume (CTV). Then, 5 mm margins were added to the CTV while creating the planning target volume (PTV). OARs included the BM, bladder, rectum, spinal cord, left femoral head, and right femoral head.

The CT and MR simulation images were transmitted into MIM 7.1.7 (MIM Software Inc., Cleveland, OH, USA). Total ABM was contoured on the FatFrac images from the L4 vertebral body to the ischial tuberosities, including the sacrum, L4-5, and pelvis [18]. The signal values of urine in the bladder (defined as the water signal) and subcutaneous fat (defined as the fat signal) of each patient were measured separately on the FatFrac images. The average of the two signal values was defined as the segmentation threshold between high-active and low-active BM.

**Fig. 1:**
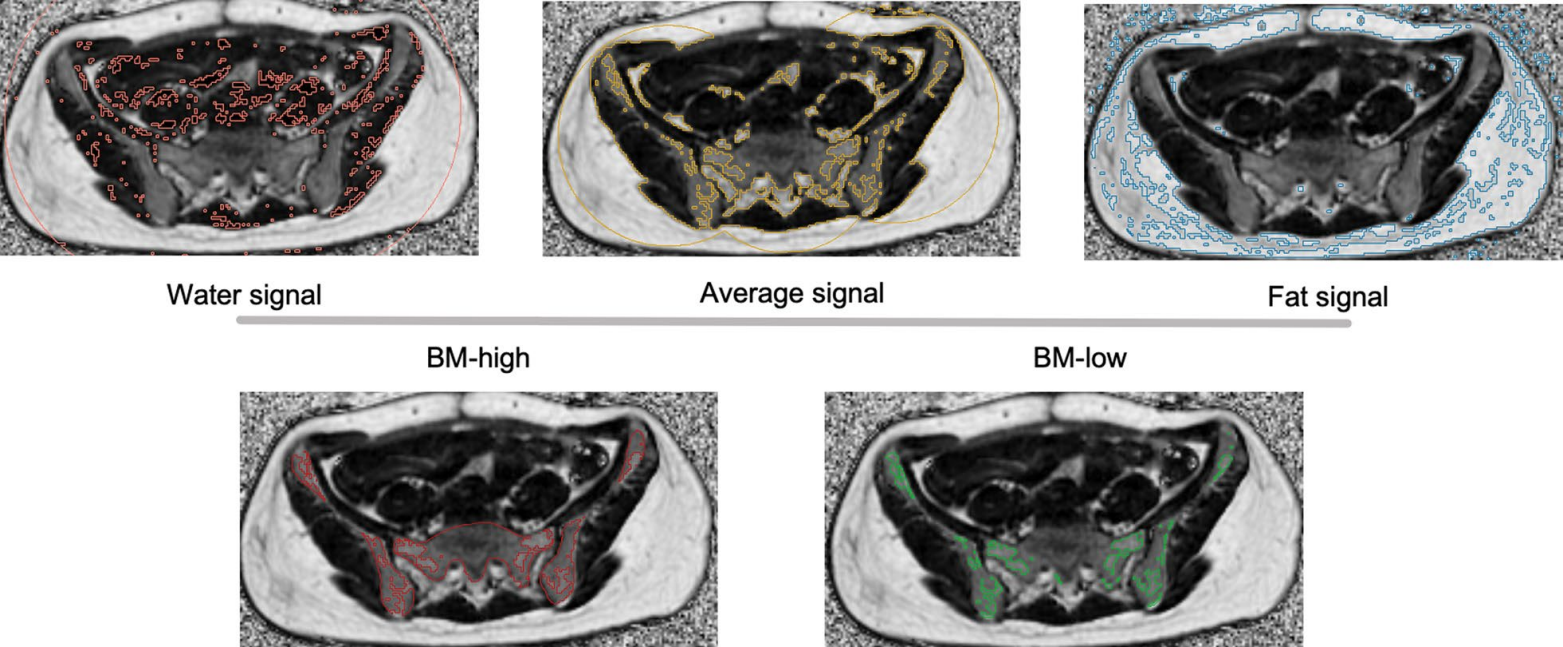
Schematic diagram of ABM segmentation

The threshold tool in MIM was used to contour high-active and low-active BM. The BM region with a signal value between the water signal and the segmentation threshold was defined as the high-active BM (ABM-_high_), and that with a signal value between the segmentation threshold and the fat signal was defined as the low-active BM (ABM-_low_) (Fig. 1). Segmented BM and ABM-_high_ regions were superimposed (rigid registered) onto the CT simulation images. Finally, the CT images with BM and ABM-_high_ structures were imported into the RayStation planning system.

### Treatment planning

Treatment plans were created in RayStation Research v10.1B (RaySearch Laboratories, Stockholm, Sweden). Four types of treatment plans were created using standardized objectives: IMRT, bone marrow sparing IMRT (IMRT-BMS), intensity modulated proton therapy (IMPT), and bone marrow sparing IMPT (IMPT-BMS). The prescription dose was 45 Gy_RBE_ in 25 fractions, with a single fraction dose of 1.8 Gy_RBE_. A generic relative biological effectiveness (RBE) value of the proton dose was set to 1.1, and the photon dose RBE value was set to 1.0. The dose constraints for OARs were as follows [19]: bladder V_30_ (the percentage of the bladder volume irradiated with more than 30 Gy) < 35%; femur head-L/R V_35_ < 5%; rectum V_40_ < 35% and V_30_ < 80%; the ABM V_10_ < 80%, V_20_ < 60%, V_30_ < 45%, and V_40_ < 30%.

For the IMRT and IMRT-BMS treatment plans, nine coplanar fields were generated. All photon treatment plans were calculated using the Monte Carlo dose calculation algorithm and the DMLC technique based on the TureBeam linear accelerator (Varian Medical Systems, Palo Alto, CA, USA) with a 6-MV photon beam. As the primary objective, we aimed to reduce the percentage volume of ABM-_high_ receiving 5–40 Gy as much as feasible when the target and other OARs were met the objectives.

**Fig. 2:**
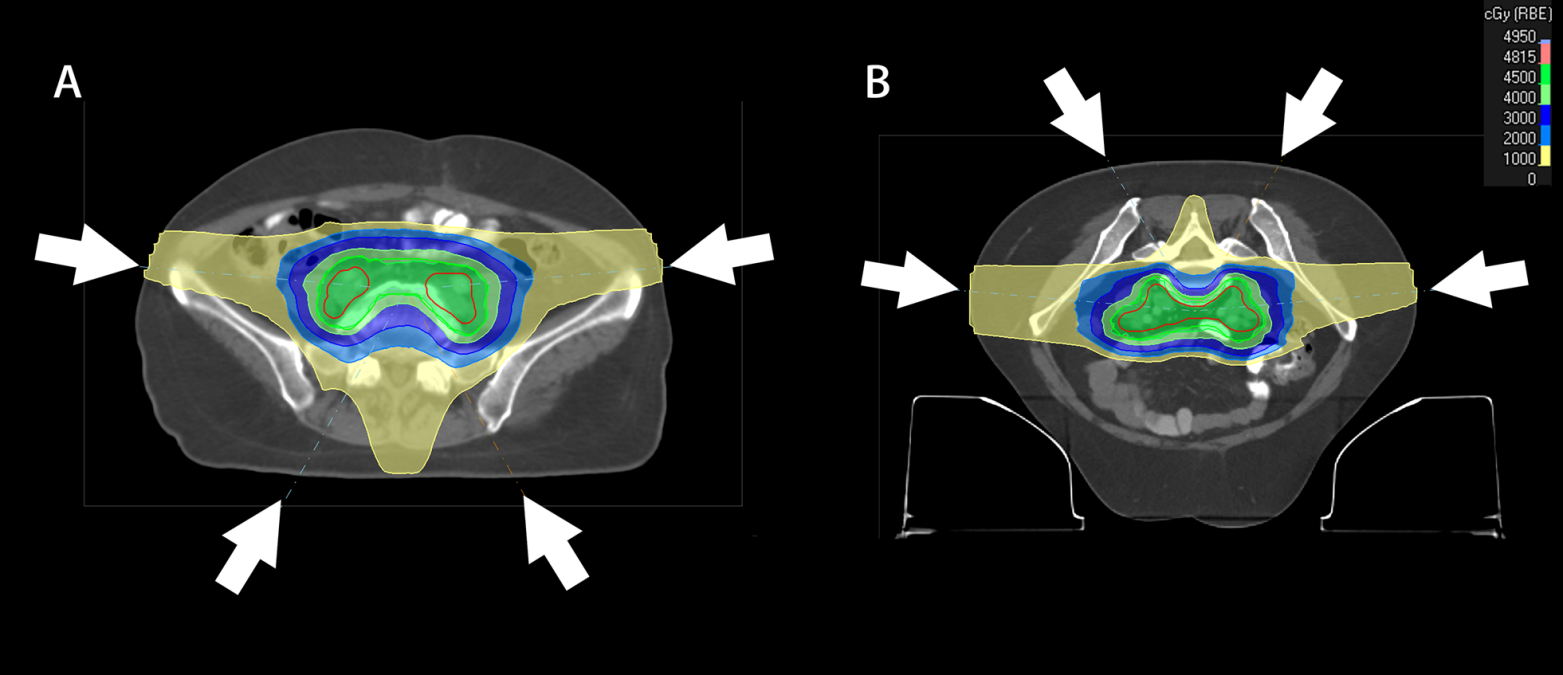
Schematic diagram of fields distribution for proton plans

For the IMPT and IMPT-BMS treatment plans, two posterior oblique beams (gantry angles of 150° and 210°) and two semi-lateral beams (gantry angles of 85° and 275°) were used for the patients in the supine position (Fig. 2A). Two anterior oblique beams (gantry angles of 30° and 330°) and two semi-lateral beams (gantry angles of 85° and 275°) were used for the patients in the prone position (Fig. 2B). The pelvic target received a dose coverage from all four beams. Robust optimization was performed for the proton treatment plans, with a 3% range and a 0.3 cm set-up uncertainty in all orthogonal directions [20]. The Monte Carlo dose calculation algorithm with 1% uncertainty was used to calculate clinical dose distributions. The proton treatment plans were generated using a machine based on ProBeam with energy layer spacing of 1 cm and spot spacing of 0.75 cm.

For IMPT and IMPT-BMS, there were nine set-up errors and three range errors creating 27 individually “perturbed doses”. For each target and OAR dose-volume objective evaluated, the static scenario (no error) and worst scenario were extracted from all the “perturbed doses”, respectively.

### Dosimetric evaluation and statistical analysis

CTV was used as the evaluation target volume for robustness optimization because PTV was used to account for delivery uncertainties that might change the intended CTV dose. For CTV, the dose of 2% and 98% volume (D_2%_, D_98%_) and the mean dose (D_mean_) were analyzed. For OARs, the V_X_ (volume percent irradiated with xGy) and the D_mean_ were analyzed. To compare the results, the coverage of each treatment plan was normalized to the same level of 95% volume of the PTV received 100% prescription dose.

For evaluating the robustness of CTV coverage in each scenario, the median value of D_98%_, D_2%_, and D_mean_ in this scenario was compared to the prescription dose (45 Gy) by conducting one-sample Wilcoxon signed-rank tests. For evaluating the robustness of OAR and ABM in each scenario, the median values of various dose-volume metrics in this scenario were compared to the IMRT values by conducting paired Wilcoxon signed-rank tests. One-sample Wilcoxon signed-rank tests were conducted to  analyze the dose volume parameters of the CTV and OARs for different body position.

Friedman’s statistical test was conducted to determine whether there were any differences among the four treatment plans. Because the results of all Friedman's tests were significant, the differences among dose parameters were assessed using paired Wilcoxon signed-rank tests. All analyses were performed using IBM SPSS Version 25 (IBM SPSS Inc, Chicago, IL), and all differences were considered to be statistically significant at *p* < 0.05.

## Results

### BM location

The mean volume of BM in all patients was 299.37 cm^3^ (191.32–417.54 cm^3^). The average volume of ABM-_high_ was 135.44 cm^3^ (19.09–267.78 cm^3^), accounting for 45.2% of the total ABM volume.

### Robustness evaluation and body position evaluation

In all perturbed scenarios, the worst values for CTV D_98%_, D_2%_, and D_mean_ were similar to the prescribed dose. All the IMPT and IMPT-BMS worst scenario values for OAR were significantly better than or not different from IMRT values. The worst scenario values of ABM and ABM-_high_ V_5_-V_40_ and D_mean_ were all significantly lower than IMRT values. The data are shown in Additional file 1: Tables S1, S2, and S3.

The dose differences in different body positions are shown in Additional file 1: Tables S4, S5, and S6. In this study, there were 15 patients in the supine position and 18 patients in the prone position. The median volume of CTV was 521.26 mL and 585.88 mL, respectively. The target coverage between different body positions was similar. Compared to that in the supine position, the left and right femoral head and rectum V_30_ of the patients in the prone position increased slightly, and the doses of other OARs were similar. In IMPT, the V_20_, V_30_ and D_mean_ of ABM and ABM-_high_ in the prone position were higher than those in the supine position.

### Dosimetric comparison between different treatment plans

#### IMRT versus IMPT

The CTV D_98%_ of the IMRT and IMPT treatment plans were similar. For IMPT, the CTV D_2%_ and D_mean_ were 0.6% and 0.4% higher than those for IMRT, respectively (Table 1). Compared to IMRT, IMPT decreased the D_mean_ of the bladder, the left, and right femoral heads, and the rectum by 17.1%, 2.6%, 2.6%, and 4.2%, respectively; the V40 of the bladder and the V_30_ of the rectum decreased by 3.2% and 8.6%, respectively (*p* < 0.001) (Table 2). The median reductions of the BM by IMPT were 0.8% for V_5_, 8.6% for V_10_, 25.3% for V_20_, 15.5% for V_30_, 9.6% for V_40_, and 12.1% for D_mean_ (all *p* < 0.05) compared to the reduction by IMRT. ABM-_high_ also decreased simultaneously by 0.6%, 8.2%, 23.5%, 15.8%, 6%, and 14.2% (*p* < 0.05) (Table 3).

**Table 1:** Dosimetric parameters of CTV for four types of plans

	OAR doses				Dose differences rate (*p* value)					
	IMRT	IMRT-BMS	IMPT	IMPT-BMS	IMRT versus IMPT	IMRT versus IMRT-BMS	IMPT versus IMPT-BMS	IMRT-BMS versus IMPT-BMS	IMRT-BMS versus IMPT	IMRT versus IMPT-BMS
CTV D_98%_										
Median	45.1	45	45.1	45.1	0%	− 0.2%	0%	0.2%	0.2%	0%
(min–max)	(44.8–45.3)	(44.8–45.3)	(44.8–45.4)	(45–45.3)	0.437	**0.001**	0.28	**< 0.001**	**0.048**	**< 0.001**
CTV D_2%_										
Median	46.3	46.6	46.8	47.3	1.1%	0.6%	1.1%	1.5%	0.4%	2.2%
(min–max)	(45.7–47.2)	(46–47.8)	(45.7–47)	(46.9–47.5)	**0.001**	**< 0.001**	**< 0.001**	**< 0.001**	0.064	**< 0.001**
CTV D_mean_										
Median	45.6	45.8	45.9	46.2	0.7%	0.4%	0.7%	0.9%	0.2%	1.3%
(min–max)	(45.3–45.9)	(45.5–46.4)	(45.6–46)	(46–46.4)	**< 0.001**	**< 0.001**	**< 0.001**	**< 0.001**	0.064	**< 0.001**

Bold fonts indicate statistical significance (*p* < 0.05)

**Table 2:** Dosimetric parameters of OAR for four types of plans

Parameter	OAR doses				Dose differences rate (*p* value)					
	IMRT	IMRT-BMS	IMPT	IMPT-BMS	IMRT versus IMPT	IMRT versus IMRT-BMS	IMPT versus IMPT-BMS	IMRT-BMS versus IMPT-BMS	IMRT-BMS versus IMPT	IMRT versus IMPT-BMS
Bladder										
V_40_										
Median	25.1	27.8	24.3	21.5	− 3.2%	10.8%	− 11.5%	− 22.7%	− 12.6%	− 14.3%
(min–max)	(10.4–37.5)	(14.5–40.2)	(10.9–36)	(10.3–36.6)	**< 0.001**	0.24	**0.001**	**< 0.001**	**< 0.001**	**< 0.001**
D_mean_										
Median	34.6	34.4	28.7	28.9	− 17.1%	− 0.6%	0.7%	− 16.0%	− 16.6%	− 16.5%
(min–max)	(28.7–37.7)	(28.8–38.2)	(20.8–34.5)	(20.5–34.6)	**< 0.001**	0.879	0.106	**< 0.001**	**< 0.001**	**< 0.001**
Femur head—L										
V_30_										
Median	9.3	6.2	5.2	5.1	− 44.1%	− 33.3%	− 1.9%	− 17.7%	− 16.1%	− 45.2%
(min–max)	(0–14.6)	(1.3–15.4)	(0.4–17.9)	(0.1–12.9)	0.503	0.126	**0.016**	**0.003**	0.469	**0.001**
D_mean_										
Median	19.5	19.6	19	18.8	− 2.6%	0.5%	− 1.1%	− 4.1%	− 3.1%	− 3.6%
(min–max)	(15.7–19.8)	(17.6–19.9)	(13.2–19.3)	(13.1–19.4)	**< 0.001**	0.081	0.427	**< 0.001**	**< 0.001**	**< 0.001**
Femur head—R										
V_30_										
Median	8.8	5.7	5.2	5.1	− 40.9%	− 35.2%	− 1.9%	− 10.5%	− 8.8%	− 42.0%
(min–max)	(0–14.6)	(1.3–17)	(0.1–20.1)	(0.1–12.5)	0.136	0.079	**0.028**	**0.004**	0.9	**< 0.001**
D_mean_										
Median	19.6	19.5	19.1	19	− 2.6%	− 0.5%	− 0.5%	− 2.6%	− 2.1%	− 3.1%
(min–max)	(17.2–19.8)	(16.1–19.8)	(11–19.3)	(10.9–19.4)	**< 0.001**	0.611	0.865	**< 0.001**	**< 0.001**	**< 0.001**
Rectum										
V_30_										
Median	71.8	82	65.6	62	− 8.6%	14.2%	− 5.5%	− 24.4%	− 20.0%	− 13.6%
(min–max)	(36.5–96.9)	(37.4–97.8)	(23.3–70.2)	(22.8–70.5)	**< 0.001**	0.299	**0.001**	**< 0.001**	**< 0.001**	**< 0.001**
V_40_										
Median	33.8	35.5	34.4	32.8	1.8%	5.0%	− 4.7%	− 7.6%	− 3.1%	− 3.0%
(min–max)	(6.9–37.1)	(8.1–40.4)	(11.3–37.1)	(9.6–37.8)	0.823	0.248	0.211	**< 0.001**	**0.001**	0.761
D_mean_										
Median	35.6	36.7	34.1	33.9	− 4.2%	3.1%	− 0.6%	− 7.6%	− 7.1%	− 4.8%
(min–max)	(28.3–38.4)	(28.6–38.9)	(23–36.1)	(23.1–35.5)	**< 0.001**	0.053	**0.03**	**< 0.001**	**< 0.001**	**< 0.001**

Bold fonts indicate statistical significance (*p* < 0.05)

**Table 3:** Dosimetric parameters of ABM and ABM-_high_ for four types of plans

Parameter	OAR doses				Dose differences rate (*p* value)					
	IMRT	IMRT-BMS	IMPT	IMPT-BMS	IMRT versus IMPT	IMRT versus IMRT-BMS	IMPT versus IMPT-BMS	IMRT-BMS versus IMPT-BMS	IMRT-BMS versus IMPT	IMRT versus IMPT-BMS
ABM										
V_5_										
Median	100	100	99.2	91.3	− 0.8%	0%	− 8.0%	− 8.7%	− 0.8%	− 8.7%
(min–max)	(94.4–100)	(94.1–100)	(90.9–100)	(85.4–98.9)	**< 0.001**	0.893	**< 0.001**	**< 0.001**	**< 0.001**	**< 0.001**
V_10_										
Median	99.9	89.2	91.3	67.2	− 8.6%	− 10.7%	− 26.4%	− 24.7%	2.4%	− 32.7%
(min–max)	(90.4–100)	(76.7–95.6)	(75.5–95.7)	(56.7–78.3)	**< 0.001**	**< 0.001**	**< 0.001**	**< 0.001**	0.514	**< 0.001**
V_20_										
Median	91.7	67.7	68.5	39.8	− 25.3%	− 26.2%	− 41.9%	− 41.2%	1.2%	− 56.6%
(min–max)	(75.9–95.6)	(58.6–77.3)	(38.3–80)	(31–50.4)	**< 0.001**	**< 0.001**	**< 0.001**	**< 0.001**	0.922	**< 0.001**
V_30_										
Median	64.6	51.1	54.6	23.4	− 15.5%	− 20.9%	− 57.1%	− 54.2%	6.8%	− 63.8%
(min–max)	(44–76.5)	(39.3–56.6)	(25.7–89.6)	(16.1–35)	**< 0.001**	**< 0.001**	**< 0.001**	**< 0.001**	0.025	**< 0.001**
V_40_										
Median	24.9	24	27.3	10.9	9.6%	− 3.6%	− 60.1%	− 54.6%	13.8%	− 56.2%
(min–max)	(13.1–38.1)	(15.5–35.5)	(10.5–38.9)	(6.4–23.1)	**0.017**	0.256	**< 0.001**	**< 0.001**	**< 0.001**	**< 0.001**
D_mean_										
Median	33.1	28.2	29.1	19.4	− 12.1%	− 14.8%	− 33.3%	− 31.2%	3.2%	− 41.4%
(min–max)	(29–39.7)	(26.4–32.7)	(23.6–35.87)	(17.7–28.5)	**< 0.001**	**< 0.001**	**< 0.001**	**< 0.001**	**0.081**	**< 0.001**
ABM-_high_										
V_5_										
Median	100	100	99.4	89.8	− 0.6%	0%	− 9.7%	− 10.2%	− 0.6%	− 10.2%
(min–max)	(92.9–100)	(92.7–100)	(87.8–100)	(80.5–98)	**< 0.001**	0.249	**< 0.001**	**< 0.001**	**< 0.001**	**< 0.001**
V_10_										
Median	99.9	89.7	91.7	63.1	− 8.2%	− 10.2%	− 31.2%	− 29.7%	2.2%	− 36.8%
(min–max)	(87–100)	(82.4–98.6)	(78–99.2)	(58.7–70.9)	**< 0.001**	**< 0.001**	**< 0.001**	**< 0.001**	**0.001**	**< 0.001**
V_20_										
Median	91.8	67.7	70.2	37.8	− 23.5%	− 26.3%	− 46.2%	− 44.2%	3.7%	− 58.8%
(min–max)	(80–97.9)	(36.3–86.4)	(47.1–93.2)	(31.2–45.1)	**< 0.001**	**< 0.001**	**< 0.001**	**< 0.001**	0.118	**< 0.001**
V_30_										
Median	66.5	50.5	56	21.7	− 15.8%	− 24.1%	− 61.3%	− 57.0%	10.9%	− 67.4%
(min–max)	(50.8–90.9)	(42.9–74.1)	(35.9–89.8)	(18.9–27.9)	**< 0.001**	**< 0.001**	**< 0.001**	**< 0.001**	**0.006**	**< 0.001**
V_40_										
Median	30.2	26.1	32	10.6	6.0%	− 13.6%	− 66.9%	− 59.4%	22.6%	− 64.9%
(min–max)	(17.7–61.1)	(17.9–40)	(16.6–55.3)	(6.6–14.7)	**0.037**	**0.04**	**< 0.001**	**< 0.001**	**< 0.001**	**< 0.001**
D_mean_										
Median	33.7	28.1	28.9	18.7	− 14.2%	− 16.6%	− 35.3%	− 33.5%	2.8%	− 44.5%
(min–max)	(29–39.7)	(26.4–32.7)	(23.6–35.87)	(17.7–28.5)	**< 0.001**	**< 0.001**	**< 0.001**	**< 0.001**	**0.081**	**< 0.001**

Bold fonts indicate statistical significance (*p* < 0.05)

#### IMRT versus IMRT-BMS

Compared to the IMRT treatment plans, the IMRT-BMS treatment plans had a similar CTV of D_98%_ (*p* > 0.05), and the IMRT-BMS plans had a slightly higher D_2%_ and D_mean_ (Table 1), which were approximately 1.1% and 0.7%, respectively (*p* < 0.05). The dose-volume parameters of all OARs in the two plans were similar (*p* > 0.05) (Table 2). The BM V_10_, V_20_, V_30_, and D_mean_ decreased by 10.7%, 26.2%, 20.9%, and 14.8%, respectively; the ABM-_high_ V_10_, V_20_, V_30_, V_40_, and D_mean_ decreased simultaneously by 10.2%, 26.3%, 24.1%, 13.6% and 16.6% (*p* < 0.001) (Table 3).

#### IMPT versus IMPT-BMS

Compared to that in the IMPT treatment plans, the CTV D_2%_ and D_mean_ in IMPT-BMS increased by 1.1% and 0.7%, respectively (*p* < 0.05) (Table 1). The V_30_ of the rectum and femoral heads decreased by 5.5% and 1.9%, respectively, in the IMPT-BMS and the V_40_ of the bladder decreased by 11.5% compared to that in the IMPT plan (*p* < 0.05) (Table 2). IMPT-BMS decreased the BM V_10_-V_40_ and the D_mean_, and the reduction range of each index was 15.5%–47.1%. The ABM-_high_ showed a similar trend, where the V_10_-V_40_ and the D_mean_ decreased by 9.7%, 31.2%, 46.2%, 61.3%, 66.9%, and 35.3%, respectively (*p* < 0.001) (Table 3).

#### IMRT-BMS versus IMPT-BMS

Compared to that in the IMRT-BMS treatment plans, the CTV D_98%_, D_2%_, and D_mean_ increased by 0.2%, 1.5%, and 0.9% in the IMPT-BMS treatment plans, respectively (Table 1). The dose-volume parameters of all OARs in the IMPT-BMS plan were lower than those in the IMRT-BMS plan, with the greatest reductions in bladder-V_40_ and rectum-V_30_ by 22.7% and 24.4%, respectively (Table 2). IMPT-BMS performed better than IMRT-BMS. The V_5_-V_40_ and D_mean_of BM and ABM-_high_ were reduced by the IMPT-BMS plans compared to their values in the IMRT-BMS plans. The reductions were as follows: 8.7% and 10.2% for V_5_, 24.7% and 29.7% for V_10_, 41.2% and 44.2% for V_20_, 54.2% and 57% for V_30_, 54.6% and 59.4% for V_40_, and 31.2% and 33.5% for D_mean_ (*p* < 0.001) (Table 3).

#### IMRT-BMS versus IMPT

Compared to the IMRT-BMS plans, the IMPT plans had a similar CTV D_2%_ and D_mean_, and the CTV D_98%_ increased in IMPT with differences within the 0.2% range (Table 1). IMPT plans significantly decreased the V_40_ of the bladder and V_30_ of the rectum by 12.6% and 20%, respectively (*p* < 0.001). ABM-_high_ V_10_-V_40_ and  D_mean_ increased in IMPT plans compared to that in IMRT-BMS, and the V_40_ had a larger increase of 22.6% (*p* < 0.001). The BM showed a similar trend of change (Table 3).

#### IMRT versus IMPT-BMS

Compared to the IMRT plans, the IMPT-BMS plans had similar CTV D_98%_ and a slightly higher CTV D_2%_ and D_mean_ (2.2% and 1.3%, respectively) (Table 1). All dose-volume parameters of OARs increased significantly using the IMPT-BMS plans (Table 2). The volume reductions are presented as follows: 45.2% and 42% for V_30_ of the left and right femoral head, 14.3% for V_40_ of the bladder, and the D_mean_ of bladder decreased from 34.6 Gy to 28.9 Gy (by 16.5%), 13.6% for V_30_ of the rectum, and the D_mean_ of the bladder decreased from 35.6 Gy to 33.9 Gy (by 4.8%) (*p* < 0.001). The V_5_-V_40_ and D_mean_ of BM decreased by 8.7%, 32.7%, 56.6%, 63.8%, 56.2%, and 41.4%, respectively. Similarly, the V_5_-V_40_ and D_mean_ of ABM-_high_ decreased by 10.2%, 36.8%, 58.8%, 67.4%, 64.9%, and 44.5% (*p* < 0.001) (Table 3).

**Fig. 3:**
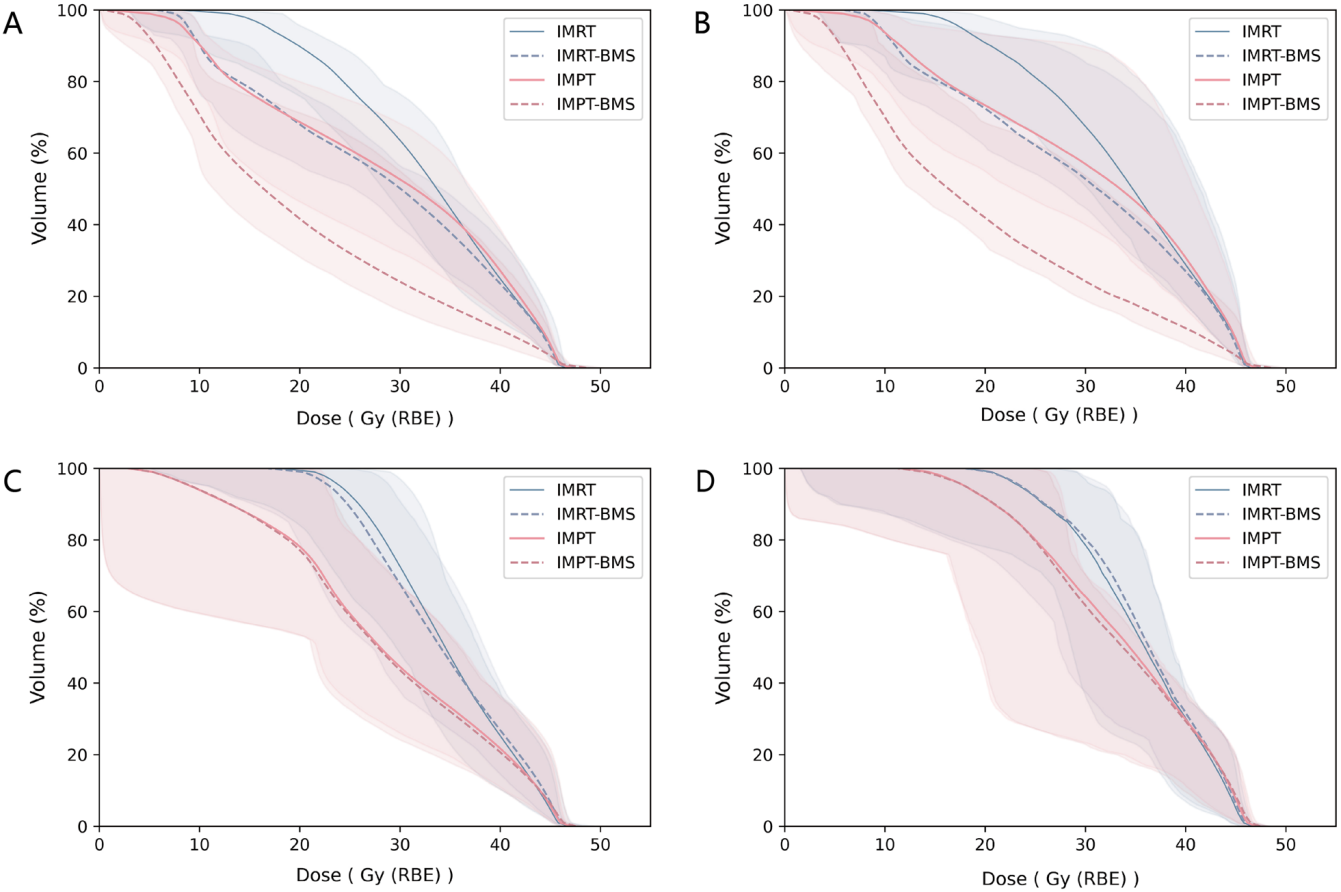
Comparison of OAR doses for ABM (**A**), ABM-_high_ (**B**), bladder (**C**), rectum (**D**) in four types of plans

Although the CTV parameters differed significantly among the four plans, the absolute difference was low, and all four types of plans had the ideal target volume coverage. IMPT-BMS showed the highest BM sparing without compromising target volume dose coverage, which significantly decreased the V_10_-V_40_ and D_mean_ of ABM-_high_. Additionally, IMRT-BMS reduced the dose for ABM-_high_ than IMRT but increased the dose in the bladder and rectum (Fig. 3).

## Discussion

In this study, we investigated the role of functional MR imaging combined with proton therapy on BMS in cervical cancer radiotherapy. Our results confirmed the feasibility of using functional MR imaging to segment high-active BM and the dosimetric advantage of proton therapy for BMS, especially PABM. Huang et al. [21] reported that 69.5% of the patients with cervical cancer who received concurrent chemoradiotherapy experienced G2 + HT. For every 1 Gy increase in the D_mean_ of PBM, the neutrophils and white blood cells of the patient decreased by 9.6/μL and 7.8/μL each week, respectively [22]. Radiation-induced lymphopenia can be associated with lower survival [23, 24]. Kumar et al. [10] reported that G4 HT was associated with PBM V_20_ > 45%. In many studies, PBM V_20_ was significantly associated with HT, and V_40_ was an important predictor of G2 + HT [12, 14]. The HT produced during concurrent chemoradiotherapy can last for at least three months after treatment is completed [25, 26].

The BM dose-volume parameter is the main index for predicting HT. However, many studies have used different prediction indices and dose-volume parameters, and no ideal radiation dose limitation for BM has been suggested yet. This can be associated with different definitions of the BM region used in various studies. Accurate identification and delineation of PABM are important. Most studies use CT bone window images to contour BM. However, no unified standard is available for BM delineation. CT images cannot distinguish BM activity, and there is a large error in delineation. PET/CT can segment actively proliferating BM regions based on tissue metabolism. However, the cost of PET/CT examination is high, the clinical popularity is poor, and the risk of radiation exists. MR functional imaging not only accurately delineates the BM region but also distinguishes and segments the ABM through the water-fat separation technique [27–29]. In another study, we reported that IDEAL IQ FatFrac imaging could be used to measure PBM fat content changes during concurrent chemoradiotherapy. The fat content of PBM increased with the increase in radiotherapy dose, especially in the PBM region close to the target region receiving high-dose irradiation (the fat fraction increased from 48.5% to 74.2%), and the change was significantly related to the HT [11]. Liang et al. [18] used the FatFrac image to contour the PBM for the IMRT-BMS plan design, which can reduce the incidence of G3 + HT from 47 to 30%. In this study, we established a method for ABM segmentation. The average value of the water and fat signal values in the FatFrac IDEAL IQ image was selected as the segmentation threshold of the ABM, and the threshold contour tool was used to manually segment the ABM. The results showed that highly active bone marrow accounted for about 45% of the total ABM volume and was dispersed, which was consistent with the results of studies by Liang and other researchers [18].

Many previous studies have been based on photon radiotherapy planning for BMS. However, some limitations are present due to the radiation dose constraints of photon beams on BM. The physical properties of protons can reduce proximal dose deposition compared with photons. The finite range and sharp distal fall-off characteristics of protons allow radiation dose to be delivered to a specific depth using the spread-out Bragg peak. The distant normal tissue receives a negligible radiation dose [16, 30]. More conformal dose distributions can be achieved due to the ability of IMPT to control the beamlet energies and intensities. The irradiation dose of BM can be further reduced based on IMRT-BMS. Dinges et al. [20] used FLT-PET/CT to identify PABM in patients with cervical cancer. Compared to IMRT, the V_10_-V_40_ reduction of ABM in the IMPT plan ranged from 32 to 60%. This was consistent with the results of this study that IMPT-BMS can reduce ABM-_high_ V_10_-V_40_ by 29.7%–59.4% compared to IMRT-BMS. Lin et al. [31] demonstrated the dosimetric advantages and clinical feasibility of pencil beam scanning, and the PBM volume was significantly reduced by 24% and 17% in 10–20 Gy low-dose irradiation. Meier et al. [32] compared IMPT and VMAT cervical cancer BMS plan and found that the D_mean_ of BM decreased from 30.76 Gy to 17.42 Gy. This was similar to the results of this study, where we found that IMPT-BMS reduced the D_mean_ of ABM-_high_ from 33.7 Gy to 18.7 Gy (by 44.5%).

By comparing four types of radiotherapy plans in this study, we found that the use of proton therapy increased the protection of PABM in the low-dose region. IMRT-BMS plans set dose constraints on ABM-_high_ based on IMRT plans, reducing ABM-_high_ V_10_-V_40_ and D_mean_. However, the ability of IMRT-BMS to modulate the dose is still limited. The dose-volume parameters of the bladder and rectum tend to increase while reducing the dose of ABM-_high_. IMPT-BMS uses the exquisite physical dose deposition characteristics of the proton beam, which can further reduce the ABM-high V_10_-V_40_ and D_mean_ based on IMRT-BMS. The D_mean_ of ABM-_high_ was as low as 18.7 Gy to achieve the best bone marrow sparing effect. IMPT-BMS can also allow significant sparing of normal tissues such as the bladder and rectum.

Since protons have a larger penumbra, normal tissues near the target may receive a higher radiation dose [16]. Xu et al. [33] reported that compared to IMRT, proton therapy performed poorly in decreasing the irradiated volume of PBM in the high-dose region (33.9–42.9 Gy), and the PBM V_40_ increased by 14.6%. In this study, IMPT ABM-_high_ V_30_ and V_40_ were 10.9% and 22.6% higher than IMRT-BMS, respectively. This was associated with the region of ABM-_high_ irradiated > 30 Gy adjacent to the target volume without dose constraints to the bone marrow. IMRT-BMS was a better choice than IMPT. After setting ABM-_high_ dose constraints in IMPT, ABM-_high_ V_30_ and V_40_ were reduced by 61.3% and 66.9%, respectively, and the D_mean_ decreased from 28.9 Gy to 18.7 Gy compared to that of IMRT-BMS.

We evaluated the feasibility of PABM delineation based on FatFrac images and bone marrow sparing with proton therapy from a dosimetric perspective only. This should be further applied to actual treatment to evaluate the effect of differences in dose reduction between different radiotherapy techniques on the risk of HT. Reliable BM radiation dose limits should be set to guide individualized radiation therapy for bone marrow sparing.

Our findings confirmed that IMPT-BMS uses its dosimetric advantage to achieve optimal BMS in radiotherapy for cervical cancer without compromising target dose volume coverage. We proposed a threshold delineation of PABM by FatFrac images, which provided a new method for PABM segmentation. MR functional imaging guidance can reduce the irradiation dose volume of PABM, and MR functional imaging combined with proton therapy can achieve optimal bone marrow sparing in radiotherapy for cervical cancer.

## Supplementary Information

**Additional file 1**. Supplementary data. **Table S1** Statistics for CTV in IMPT and IMRT-BMS in static and worst scenarios. **Table S2** Statistics for OAR in IMPT and IMRT-BMS in static and worst scenarios. **Table S3** Statistics for PABM in IMPT and IMRT-BMS in static and worst scenarios. **Table S4** Dose differences in different body positions of CTV. **Table S5** Dose differences in different body positions of OAR. **Table S6** Dose differences in different body positions of ABM and ABM-_high_.

## Data Availability

All data obtained during the current study are available from the corresponding author on reasonable request.

## Abbreviations

PABM: Pelvic active bone marrow
MR: Magnetic resonance
ABM: Active bone marrow
ABM-_high_: High-active bone marrow
IMRT: Intensity modulated photon therapy
IMRT-BMS: Bone marrow sparing IMRT
IMPT: Intensity modulated proton therapy
IMPT-BMS: Bone marrow sparing IMPT
HT: Hematological toxicity
BM: Bone marrow
BMS: Bone marrow sparing
FatFrac IDEAL IQ: Fat fraction
OAR: Organ at risk
GTV: Gross tumor volume
CTV: Clinical target volume
PTV: Planning target volume
ABM-_low_: Low-active BM
RBE: Relative biological effectiveness
