# Supplementary material for: Dosimetric evaluation of bone marrow sparing in proton radiotherapy for cervical cancer guided by MR functional imaging
Source: Radiat Oncol. 2022 Dec 14;17:207. doi: 10.1186/s13014-022-02175-3 (PMC9753489; doi:10.1186/s13014-022-02175-3)
Supplement: Supplementary file 1 — Additional file 1. Supplementary data. Table S1 Statistics for CTV in IMPT and IMRT-BMS in static and worst scenarios. Table S2 Statistics for OAR in IMPT and IMRT-BMS in static and worst scenarios. Table S3 Statistics for PABM in IMPT and IMRT-BMS in static and worst scenarios. Table S4 Dose differences in different body positions of CTV. Table S5 Dose differences in different body positions of OAR. Table S6 Dose differences in different body positions of ABM and ABM-high. [file 13014_2022_2175_MOESM1_ESM.docx]

Table 1 Statistics for CTV in IMPT and IMRT-BMS in static and worst scenarios.

|  |  | IMRT | IMPT  static | IMPT-BMS static | IMPT  worst | IMPT-BMS worst |
| --- | --- | --- | --- | --- | --- | --- |
| CTV D_98%_ | median | 45.1 | 45.1 | 45.1 | 44.9 | 44.9 |
|  | (min-max) | (44.8-45.3) | (44.8-45.4) | (45-45.3) | (44.6-45) | (44.8-45.4) |
|  | *p*-Value | **0.046** | **< 0.001** | **< 0.001** | **< 0.001** | **< 0.001** |
| CTV D_2%_ | median | 46.3 | 46.8 | 47.3 | 46.7 | 47.3 |
|  | (min-max) | (45.7-47.2) | (45.7-47) | (46.9-47.5) | (45.7-47) | (46.2-47.5) |
|  | *p*-Value | **< 0.001** | **< 0.001** | **< 0.001** | **< 0.001** | **< 0.001** |
| CTV D_mean_ | median | 45.6 | 45.9 | 46.2 | 45.8 | 46.1 |
|  | (min-max) | (45.3-45.9) | (45.6-46) | (46-46.4) | (45.2-45.9) | (44.3-46.3) |
|  | *p*-Value | **< 0.001** | **< 0.001** | **< 0.001** | **< 0.001** | **< 0.001** |

Table 2 Statistics for OAR in IMPT and IMRT-BMS in static and worst scenarios.

|  |  |  | IMRT | IMPT  static | IMPT-BMS static | IMPT  worst | IMPT-BMS worst |
| --- | --- | --- | --- | --- | --- | --- | --- |
| Bladder | V_40_ | median | 25.1 | 24.3 | 21.5 | 28.2 | 25.2 |
|  |  | (min-max) | (10.4-37.5) | (10.9-36) | (10.3-36.6) | (12.4-46.1) | (11.4-47.9) |
|  |  | *p*-Value | Reference | **< 0.001** | **< 0.001** | **0.034** | 0.607 |
|  | D | median | 34.6 | 28.7 | 28.9 | 29.3 | 29.5 |
|  |  | (min-max) | (28.7-37.7) | (20.8-34.5) | (20.5-34.6) | (22.6-37) | (22.4-37.6) |
|  |  | *p*-Value | Reference | **< 0.001** | **< 0.001** | **< 0.001** | **< 0.001** |
| Femur head - L | V_30_ | median | 9.3 | 5.2 | 5.1 | 15.3 | 13.9 |
|  |  | (min-max) | (0-14.6) | (0.4-17.9) | (0.1-12.9) | (3.9-27.5) | (1.7-22.7) |
|  |  | *p*-Value | Reference | 0.503 | **0.001** | **< 0.001** | **< 0.001** |
|  | D_mean_ | median | 19.5 | 19 | 18.8 | 21.5 | 20.9 |
|  |  | (min-max) | (15.7-19.8) | (13.2-19.3) | (13.1-19.4) | (15.6-22.9) | (15.4-21.9) |
|  |  | *p*-Value | Reference | **< 0.001** | **< 0.001** | **< 0.001** | **< 0.001** |
| Femur head - R | V_30_ | median | 8.8 | 5.2 | 5.1 | 13.1 | 12.7 |
|  |  | (min-max) | (0-14.6) | (0.1-20.1) | (0.1-12.5) | (1.7-29) | (1.8-22.1) |
|  |  | *p*-Value | Reference | 0.136 | **< 0.001** | **< 0.001** | **< 0.001** |
|  | D_mean_ | median | 19.6 | 19.1 | 19 | 21 | 20.7 |
|  |  | (min-max) | (17.2-19.8) | (11-19.3) | (10.9-19.4) | (13.5-22.8) | (13.3-22) |
|  |  | *p*-Value | Reference | **< 0.001** | **< 0.001** | **< 0.001** | **0.001** |
| Rectum | V_30_ | median | 71.8 | 65.6 | 62 | 73.8 | 71.4 |
|  |  | (min-max) | (36.5-96.9) | (23.3-70.2) | (22.8-70.5) | (25.6-79.1) | (25.5-81.1) |
|  |  | *p*-Value | Reference | **< 0.001** | **< 0.001** | 0.064 | **0.01** |
|  | V_40_ | median | 33.8 | 34.4 | 32.8 | 41.5 | 40.4 |
|  |  | (min-max) | (6.9-37.1) | (11.3-37.1) | (9.6-37.8) | (13.5-48.6) | (11.6-49.3) |
|  |  | *p*-Value | Reference | 0.823 | 0.761 | **< 0.001** | **< 0.001** |
|  | D_mean_ | median | 35.6 | 34.1 | 33.9 | 35.8 | 35.5 |
|  |  | (min-max) | (28.3-38.4) | (23-36.1) | (23.1-35.5) | (23.8-37.5) | (23.8-37.8) |
|  |  | *p*-Value | Reference | **< 0.001** | **< 0.001** | 0.809 | 0.224 |

Table 3 Statistics for PABM in IMPT and IMRT-BMS in static and worst scenarios.

|  |  |  | IMRT | IMPT  static | IMPT-BMS  static | IMPT  worst | IMPT-BMS  worst |
| --- | --- | --- | --- | --- | --- | --- | --- |
| ABM | V_5_ | median | 100 | 99.2 | 91.3 | 99.5 | 92.8 |
|  |  | (min-max) | (94.4-100) | (90.9-100) | (85.4-98.9) | (92-100) | (87.2-98.7) |
|  |  | *p*-Value | Reference | **< 0.001** | **< 0.001** | **< 0.001** | **< 0.001** |
|  | V_10_ | median | 99.9 | 91.3 | 67.2 | 92.5 | 73 |
|  |  | (min-max) | (90.4-100) | (75.5-95.7) | (56.7-78.3) | (79.8-96.7) | (62.4-78.5) |
|  |  | *p*-Value | Reference | **< 0.001** | **< 0.001** | **< 0.001** | **< 0.001** |
|  | V_20_ | median | 91.7 | 68.5 | 39.8 | 72.6 | 47.1 |
|  |  | (min-max) | (75.9-95.6) | (38.3-80) | (31-50.4) | (52.7-82.9) | (36.7-54.7) |
|  |  | *p*-Value | Reference | **< 0.001** | **< 0.001** | **< 0.001** | **< 0.001** |
|  | V_30_ | median | 64.6 | 54.6 | 23.4 | 60.2 | 29.6 |
|  |  | (min-max) | (44-76.5) | (25.7-89.6) | (16.1-35) | (38.3-74.6) | (21.2-38.2) |
|  |  | *p*-Value | Reference | **< 0.001** | **< 0.001** | **0.017** | **< 0.001** |
|  | V_40_ | median | 24.9 | 27.3 | 10.9 | 32.1 | 15.4 |
|  |  | (min-max) | (13.1-38.1) | (10.5-38.9) | (6.4-23.1) | (17.8-44.5) | (9.4-21.7) |
|  |  | *p*-Value | Reference | **0.017** | **< 0.001** | **< 0.001** | **< 0.001** |
|  | D_mean_ | median | 33.1 | 29.1 | 19.4 | 30.8 | 21.6 |
|  |  | (min-max) | (27.4-35.7) | (21.6-32.8) | (16.3-29.2) | (23.4-34.1) | (18.1-23.8) |
|  |  | *p*-Value | Reference | **< 0.001** | **< 0.001** | **< 0.001** | **< 0.001** |
| ABM-high | V_5_ | median | 100 | 99.4 | 89.8 | 99.6 | 92.7 |
|  |  | (min-max) | (92.9-100) | (87.8-100) | (80.5-98) | (90.6-100) | (84.9-98.9) |
|  |  | *p*-Value | Reference | **< 0.001** | **< 0.001** | **< 0.001** | **< 0.001** |
|  | V_10_ | median | 99.9 | 91.7 | 63.1 | 93.8 | 70.8 |
|  |  | (min-max) | (87-100) | (78-99.2) | (58.7-70.9) | (82.1-99.5) | (66.8-92.5) |
|  |  | *p*-Value | Reference | **< 0.001** | **< 0.001** | **< 0.001** | **< 0.001** |
|  | V_20_ | median | 91.8 | 70.2 | 37.8 | 74.4 | 46.6 |
|  |  | (min-max) | (80-97.9) | (47.1-93.2) | (31.2-45.1) | (56.4-94.5) | (39.9-77375) |
|  |  | *p*-Value | Reference | **< 0.001** | **< 0.001** | **< 0.001** | **< 0.001** |
|  | V_30_ | median | 66.5 | 56 | 21.7 | 63.1 | 28.6 |
|  |  | (min-max) | (50.8-90.9) | (35.9-89.8) | (18.9-27.9) | (45.7-91.5) | (24.8-59.1) |
|  |  | *p*-Value | Reference | **< 0.001** | **< 0.001** | 0.127 | **< 0.001** |
|  | V_40_ | median | 30.2 | 32 | 10.6 | 37 | 15.5 |
|  |  | (min-max) | (17.7-61.1) | (16.6-55.3) | (6.6-14.7) | (20.5-65) | (10.5-37.5) |
|  |  | *p*-Value | Reference | **0.037** | **< 0.001** | **0.01** | **< 0.001** |
|  | D_mean_ | median | 33.7 | 28.9 | 18.7 | 31.6 | 21.5 |
|  |  | (min-max) | (29-39.7) | (23.6-35.9) | (17.7-28.5) | (25.4-36.7) | (19.9-32) |
|  |  | *p*-Value | Reference | **< 0.001** | **< 0.001** | **0.001** | **< 0.001** |

Table 4 Dose differences in different body positions of CTV

|  |  | IMRT |  | IMRT-BMS |  | IMPT |  | IMPT-BMS |  |
| --- | --- | --- | --- | --- | --- | --- | --- | --- | --- |
|  |  | supine | prone | supine | prone | supine | prone | supine | prone |
| D_98%_ | median | 45 | 45.1 | 45 | 45.1 | 45.1 | 45 | 45.1 | 45.1 |
|  | (min-max) | (44.8-45.3) | (44.8-45.3) | (44.8-45.3) | (44.9-45.2) | (44.8-45.2) | (45-45.4) | (45-45.3) | (45-45.3) |
|  | *p*-Value | 0.12 |  | 0.051 |  | **0.014** |  | 0.638 |  |
| D_2%_ | median | 46.3 | 46.1 | 46.4 | 46.7 | 46.8 | 46.8 | 47.1 | 47.3 |
|  | (min-max) | (45.8-47.2) | (45.7-46.7) | (46-46.8) | (46.4-47.8) | (46.5-46.8) | (45.7-47) | (46.9-47.5) | (47.1-47.5) |
|  | *p*-Value | 0.089 |  | **0.001** |  | **0.047** |  | **0.005** |  |
| D_mean_ | median | 45.6 | 45.6 | 45.7 | 45.9 | 46 | 46 | 46.1 | 46.2 |
|  | (min-max) | (45.4-45.9) | (45.3-45.8) | (45.5-45.9) | (45.7-46.4) | (45.8-46) | (45.5-46) | (46-46.3) | (46.1-46.4) |
|  | *p*-Value | 0.24 |  | **< 0.001** |  | 0.426 |  | **0.006** |  |

Table 5 Dose differences in different body positions of OAR

|  |  |  | IMRT |  | IMRT-BMS |  | IMPT |  | IMPT-BMS |  |
| --- | --- | --- | --- | --- | --- | --- | --- | --- | --- | --- |
|  |  |  | supine | prone | supine | prone | supine | prone | supine | prone |
| Bladder | V_40_ | median | 24.8 | 26.5 | 26.7 | 27.9 | 20.2 | 25.3 | 20 | 23.7 |
|  |  | (min-max) | (10.4-35.4) | (11.6-37.5) | (14.5-36.8) | (15.7-40.2) | (11.6-33.4) | (10.9-36) | (10.8-32.9) | (10.3-36.6) |
|  |  | *p*-Value | 0.206 |  | 0.448 |  | 0.159 |  | 0.226 |  |
|  | D_mean_ | median | 34.8 | 34.2 | 34.4 | 33.6 | 28.7 | 28.2 | 29.4 | 27.6 |
|  |  | (min-max) | (31.3-37.7) | (28.7-37.3) | (29.9-37.5) | (28.8-38.2) | (24.6-34.5) | (20.8-34.5) | (24.7-34.6) | (20.5-33.3) |
|  |  | *p*-Value | 0.255 |  | 0.745 |  | 0.47 |  | 0.247 |  |
| Femur head-L | V_30_ | median | 5.1 | 9.9 | 5.2 | 6.3 | 5 | 7.4 | 4.6 | 5.4 |
|  |  | (min-max) | (0-12.4) | (4.9-14.6) | (1.3-14) | (4.9-15.4) | (0.4-15.9) | (5-17.9) | (0.1-11.1) | (4.8-12.9) |
|  |  | *p*-Value | **0.017** |  | **0.043** |  | **0.014** |  | **0.013** |  |
|  | D_mean_ | median | 19.6 | 19.4 | 19.6 | 19.7 | 19 | 19 | 18.8 | 18.6 |
|  |  | (min-max) | (15.7-19.8) | (18.7-19.7) | (17.9-19.8) | (17.6-19.9) | (13.2-19.3) | (16.1-19.3) | (13.1-19.4) | (16.6-19.2) |
|  |  | *p*-Value | 0.899 |  | 0.426 |  | 0.492 |  | 0.745 |  |
| Femur head-R | V_30_ | median | 5.8 | 9.3 | 5.3 | 6 | 4.8 | 7.7 | 4.7 | 5.3 |
|  |  | (min-max) | (0-11.2) | (3.1-14.6) | (1.3-12) | (3.1-17) | (0.1-10.7) | (4.3-20.1) | (0.1-10.8) | (3.8-12.5) |
|  |  | *p*-Value | **0.011** |  | 0.063 |  | **0.009** |  | **0.007** |  |
|  | D_mean_ | median | 19.6 | 19.5 | 19.5 | 19.5 | 19.1 | 18.7 | 19 | 18.6 |
|  |  | (min-max) | (17.2-19.8) | (18.9-19.7) | (16.1-19.8) | (18.4-19.8) | (11-19.3) | (16.8-19.2) | (10.9-19.3) | (14.2-19.4) |
|  |  | *p*-Value | 0.678 |  | 0.942 |  | 0.426 |  | 0.426 |  |
| Rectum | V_30_ | median | 77.4 | 74 | 87.2 | 80.9 | 64.4 | 66.6 | 65.3 | 61.2 |
|  |  | (min-max) | (36.5-96.9) | (58.6-90.5) | (37.4-97.6) | (48.9-97.8) | (23.3-69.8) | (59.9-70.2) | (22.8-70.5) | (58.2-66.3) |
|  |  | *p*-Value | **0.006** |  | 0.347 |  | 0.148 |  | **0.011** |  |
|  | V_40_ | median | 34.9 | 33.5 | 35.5 | 35.7 | 34.4 | 34.7 | 35.2 | 32.1 |
|  |  | (min-max) | (6.9-37.1) | (15.6-35.4) | (8.1-38.5) | (14.9-40.4) | (11.3-37.1) | (15.7-36.1) | (9.6-37.8) | (14-36.5) |
|  |  | *p*-Value | 0.148 |  | 0.885 |  | 0.899 |  | 0.338 |  |
|  | D_mean_ | median | 36.8 | 35.2 | 36.7 | 36.7 | 33.4 | 34.8 | 33.4 | 34.6 |
|  |  | (min-max) | (28.3-38.4) | (30-38) | (29.1-38.9) | (28.6-38.5) | (23-35) | (30.3-36.1) | (23.1-35.5) | (29-35.5) |
|  |  | *p*-Value | **0.031** |  | 0.515 |  | **0.019** |  | 0.347 |  |

Table 6 Dose differences in different body positions of ABM and ABM-_high_

|  |  |  | IMRT |  | IMRT-BMS |  | IMPT |  | IMPT-BMS |  |
| --- | --- | --- | --- | --- | --- | --- | --- | --- | --- | --- |
|  |  |  | supine | prone | supine | prone | supine | prone | supine | prone |
| ABM | V_5_ | median | 100 | 100 | 100 | 100 | 99.5 | 98.8 | 91.4 | 90.8 |
|  |  | (min-max) | (94.4-100) | (95.5-100) | (94.1-100) | (99.7-100) | (90.9-100) | (92.4-100) | (85.4-98.9) | (85.9-95.9) |
|  |  | *p*-Value | 0.861 |  | 0.717 |  | **0.024** |  | 0.12 |  |
|  | V_10_ | median | 99.9 | 99.7 | 88.9 | 91.2 | 90.6 | 92 | 68.4 | 65.4 |
|  |  | (min-max) | (91.5-100) | (90.4-100) | (76.7-94.1) | (84.4-95.6) | (75.5-93.4) | (79.8-95.7) | (62.5-76.5) | (56.7-78.3) |
|  |  | *p*-Value | 0.062 |  | 0.138 |  | 0.083 |  | 0.219 |  |
|  | V_20_ | median | 89.8 | 92.8 | 69.7 | 66.6 | 63.7 | 71.5 | 39.2 | 41.2 |
|  |  | (min-max) | (76.6-95.4) | (75.9-95.6) | (61-73.2) | (58.6-77.3) | (38.3-74.8) | (58.6-80) | (33.1-50.4) | (31-47.2) |
|  |  | *p*-Value | 0.12 |  | 0.053 |  | **0.007** |  | 0.233 |  |
|  | V_30_ | median | 64.6 | 63.2 | 51.6 | 48.9 | 48.4 | 56.2 | 23.4 | 23.3 |
|  |  | (min-max) | (44-76.5) | (46.2-74.1) | (39.3-56.6) | (40.2-56.3) | (25.7-89.6) | (43.8-68.3) | (19.5-35) | (16.1-30.3) |
|  |  | *p*-Value | 0.426 |  | 0.112 |  | **0.033** |  | 0.942 |  |
|  | V_40_ | median | 24.5 | 25.8 | 24.1 | 23.3 | 27 | 28 | 11.4 | 10.4 |
|  |  | (min-max) | (13.1-36.2) | (15.5-38.1) | (15.5-35.5) | (17.1-28.8) | (10.5-35.6) | (21.4-38.9) | (7.3-23.1) | (6.4-13.5) |
|  |  | *p*-Value | 0.47 |  | 0.731 |  | 0.159 |  | 0.164 |  |
|  | D_mean_ | median | 33.1 | 33 | 28.7 | 28 | 27.6 | 29.5 | 19.6 | 19.3 |
|  |  | (min-max) | (27.4-35.1) | (28.8-35.7) | (24.8-30.9) | (25-30.4) | (21.6-31) | (26-32.7) | (17.7-29.2) | (16.2-21.3) |
|  |  | *p*-Value | 0.704 |  | 0.096 |  | **0.019** |  | 0.638 |  |
| ABM-_high_ | V_5_ | median | 100 | 100 | 100 | 100 | 99.4 | 99.1 | 90.2 | 89 |
|  |  | (min-max) | (92.9-100) | (100-100) | (92.7-100) | (94.5-100) | (87.8-100) | (96-100) | (80.5-98) | (84.8-95.3) |
|  |  | *p*-Value | 0.273 |  | 0.524 |  | 0.563 |  | 0.133 |  |
|  | V_10_ | median | 99.9 | 99.8 | 88.4 | 90.2 | 90.2 | 94.1 | 65.2 | 61.9 |
|  |  | (min-max) | (87-100) | (94.2-100) | (82.4-95.6) | (83.3-98.6) | (78-98) | (84.7-99.2) | (60.7-70.9) | (58.7-70.4) |
|  |  | *p*-Value | 0.598 |  | 0.613 |  | 0.053 |  | **0.01** |  |
|  | V_20_ | median | 90.4 | 93.4 | 69.3 | 66.3 | 64.6 | 74.9 | 37.9 | 37.8 |
|  |  | (min-max) | (80-96.7) | (82.4-97.9) | (63-86.4) | (36.3-85.6) | (47.1-85.8) | (64.4-93.2) | (31.2-45.1) | (35.4-43.1) |
|  |  | *p*-Value | 0.057 |  | 0.053 |  | **0.005** |  | 0.745 |  |
|  | V_30_ | median | 65.3 | 66.7 | 51.2 | 49.8 | 53.3 | 61 | 21.9 | 21.3 |
|  |  | (min-max) | (51.1-85.8) | (50.8-90.9) | (46-74.1) | (42.9-67.9) | (35.9-77.1) | (49.7-89.8) | (19.8-27.9) | (18.9-25.4) |
|  |  | *p*-Value | 0.914 |  | 0.129 |  | **0.03** |  | 0.158 |  |
|  | V_40_ | median | 30.2 | 30.3 | 26.1 | 25.9 | 30.8 | 33.5 | 10.8 | 9.7 |
|  |  | (min-max) | (17.7-40.3) | (17.7-61.1) | (17.9-38.5) | (19.4-40) | (16.6-43.4) | (21.8-55.3) | (6.9-14.7) | (6.6-14.4) |
|  |  | *p*-Value | 0.426 |  | 0.942 |  | 0.096 |  | 0.055 |  |
|  | D_mean_ | median | 34.5 | 33.5 | 28.1 | 28.2 | 27.1 | 29.5 | 19.3 | 18.5 |
|  |  | (min-max) | (29-36.3) | (30.2-39.7) | (26.9-32.7) | (26.4-32.2) | (23.6-33) | (28-35.7) | (17.8-28.5) | (17.7-19.9) |
|  |  | *p*-Value | 0.814 |  | 0.492 |  | **0.004** |  | 0.071 |  |
